# [2,7-Dibut­oxy-8-(4-fluoro­benzo­yl)naphthalen-1-yl](4-fluoro­phen­yl)methanone

**DOI:** 10.1107/S1600536812044923

**Published:** 2012-11-03

**Authors:** Daichi Hijikata, Kosuke Sasagawa, Sayaka Yoshiwaka, Akiko Okamoto, Noriyuki Yonezawa

**Affiliations:** a Department of Organic and Polymer Materials Chemistry, Tokyo University of Agriculture & Technology (TUAT), Koganei, Tokyo 184.8588, Japan

## Abstract

In the title compound, C_32_H_30_F_2_O_4_, the benzene rings of the benzoyl groups make dihedral angles of 74.55 (6) and 74.39 (7)° with the naphthalene ring system. In the crystal, intra- and inter­molecular C—H⋯π inter­actions are observed between the but­oxy group and the aromatic rings. There are also C—H⋯F hydrogen bonds present that link the mol­ecules into chains propagating along [010].

## Related literature

For the electrophilic aromatic aroylation of 2,7-dimeth­oxy­naphthalene, see: Okamoto & Yonezawa (2009 ▶); Okamoto *et al.* (2011 ▶, 2012 ▶). For the crystal structures of similar compounds, see: Sasagawa *et al.* (2011 ▶); Watanabe *et al.* (2010 ▶).
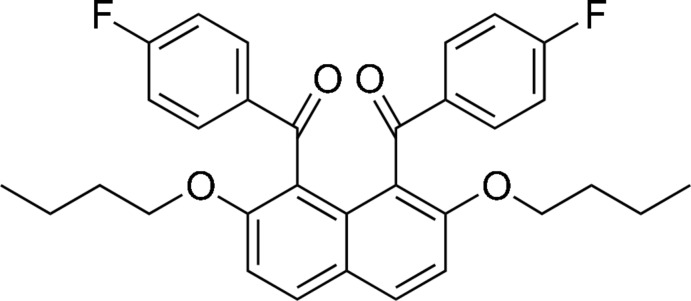


## Experimental

### 

#### Crystal data

C_32_H_30_F_2_O_4_
*M* *_r_* = 516.56
Monoclinic, 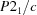

*a* = 8.26012 (15) Å
*b* = 20.2309 (4) Å
*c* = 16.5268 (3) Å
β = 99.918 (1)°
*V* = 2720.51 (9) Å^3^
*Z* = 4
Cu *K*α radiation
μ = 0.75 mm^−1^
*T* = 193 K
0.60 × 0.50 × 0.50 mm

#### Data collection

Rigaku R-AXIS RAPID diffractometer
Absorption correction: numerical (*NUMABS*; Higashi, 1999 ▶) *T*_min_ = 0.661, *T*_max_ = 0.705
50395 measured reflections
4971 independent reflections
4715 reflections with *I* > 2σ(*I*)
*R*_int_ = 0.047

#### Refinement

*R*[*F* ^2^ > 2σ(*F* ^2^)] = 0.045
*wR*(*F* ^2^) = 0.116
*S* = 1.03
4971 reflections
346 parameters
H-atom parameters constrained
Δρ_max_ = 0.35 e Å^−3^
Δρ_min_ = −0.41 e Å^−3^

### 

Data collection: *PROCESS-AUTO* (Rigaku, 1998 ▶); cell refinement: *PROCESS-AUTO*; data reduction: *PROCESS-AUTO*; program(s) used to solve structure: *Il Milione* (Burla *et al.*, 2007 ▶); program(s) used to refine structure: *SHELXL97* (Sheldrick, 2008 ▶); molecular graphics: *ORTEPIII* (Burnett & Johnson, 1996 ▶); software used to prepare material for publication: *SHELXL97*.

## Supplementary Material

Crystal structure: contains datablock(s) I, global. DOI: 10.1107/S1600536812044923/su2520sup1.cif

Structure factors: contains datablock(s) I. DOI: 10.1107/S1600536812044923/su2520Isup2.hkl

Supplementary material file. DOI: 10.1107/S1600536812044923/su2520Isup3.cml

Additional supplementary materials:  crystallographic information; 3D view; checkCIF report

## Figures and Tables

**Table 1:** Hydrogen-bond geometry (Å, °) *Cg*1 and *Cg*2 are the centroids of the C12–C17 and C5–C10 rings, respectively.

*D*—H⋯*A*	*D*—H	H⋯*A*	*D*⋯*A*	*D*—H⋯*A*
C27—H27*B*⋯*Cg*1	0.99	2.79	3.7754 (19)	175
C26—H26*A*⋯*Cg*2^i^	0.99	2.54	3.4239 (16)	145
C3—H3⋯F2^ii^	0.95	2.50	3.4408 (17)	169

Symmetry codes: (i) 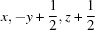; (ii) 
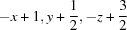
.

**Fig. 1.**
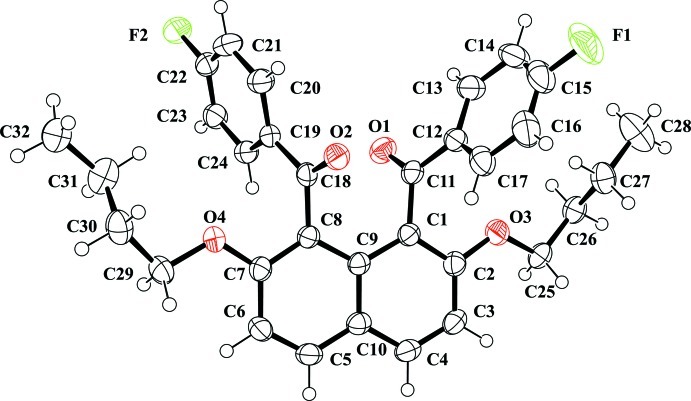
The molecular structure of title molecule, with the atom numbering. The displacement ellipsoids are drawn at the 50% probability level.

**Fig. 2.**
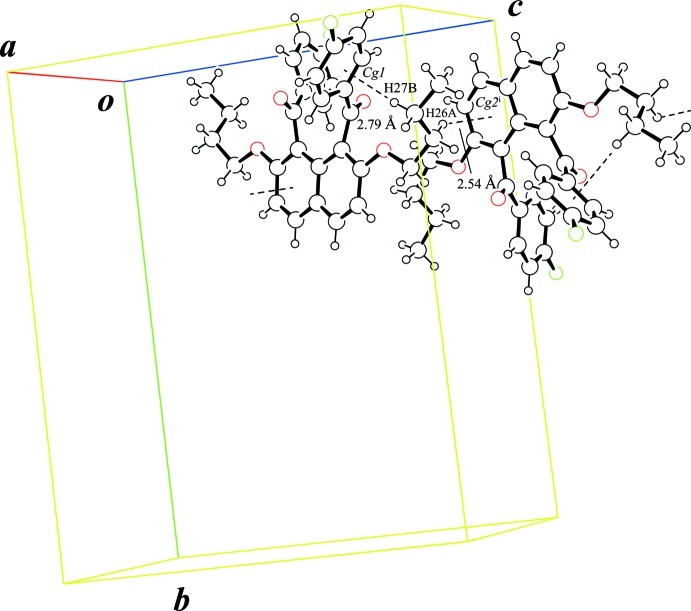
A partial crystal packing diagram of the title compound. The intra- and intermolecular C—H···π interactions are shown as dashed lines.

## supplementary crystallographic information

### Comment

In the course of our studies on electrophilic aromatic aroylation of 2,7-dimethoxynaphthalene, *peri*-aroylnaphthalene compounds have proven to be formed regioselectively with the aid of suitable acidic mediators (Okamoto & Yonezawa, 2009; Okamoto, Mitsui *et al.*, 2011). In one application, the authors have integrated the resulting molecular unit to a poly(ether ketone) backbone *via* nucleophilic aromatic substitution polycondensation (Okamoto *et al.*, 2012). The poly(ether ketone)s composed of 1,8-diaroylenenaphthalene units show unique thermal properties and solubility for organic solvents. These curious features of the polymers can be explained on the basis of the structural features of the 1,8-diaroylene naphthalene units. Under these circumstances, the authors have undertaken the X-ray crystal structural study of several 1,8-diaroylated naphthalene analogues, exemplified by (2,7-dimethoxynaphthalene-1,8-diyl)bis(4-fluorophenyl)dimethanone (Watanabe *et al.*, 2010) and [8-(4-butoxybenzoyl)-2,7-dimethoxy- naphthalen-1-yl](4-butoxyphenyl)-methanone (Sasagawa *et al.*, 2011). These molecules have essentially the same non-coplanar features. The aroyl groups at the 1,8-positions of the naphthalene rings in these molecules are twisted and bonded in an almost perpendicular fashion, but the benzene ring moieties of the aroyl groups tilt slightly toward the *exo* sides of the naphthalene rings. As a part of our continuous studies on the molecular structures of this kind of homologous molecules, the X-ray crystal structure of the title compound is presented herein.

The molecular structure of the title compound is displayed in Fig. 1. Two benzoyl groups at the 1,8-positions of the naphthalene ring are situated in opposite directions, with an *anti* orientation. The benzene rings of the benzoyl groups make dihedral angles with the naphthalene ring system of 74.55 (6) and 74.39 (7)°, respectively. The dihedral angle between these benzene rings is 44.61 (8)°.

In the crystal structure, the molecular packing of the title compound is stabilized mainly by two types of C—H···π interactions: a) an intramolecular C—H···π interaction between the benzene ring of the aroyl group (C12—C17; *Cg*1) and one methylene H atom (H27B) of the butoxy group (C27—H27B···*Cg1*= 2.79 Å; Fig. 2 and Table 1); and b) an intermolecular C—H···π interaction between the centroid of the C5—C10 ring (*Cg*2) and one methylene H atom (H26A) of the butoxy group (C26—H26A···*Cg2^i^*= 2.54 Å; Fig. 2 and Table 1). There is also a C-H···F hydrogen bond present (Table 1) that links the molecules to form chains propagating along the b axis direction.

### Experimental

The title compound was prepared by treating a mixture of 2,7-dibutoxynaphthalene (3.0 mmol, 817 mg) and 4-fluorobenzoic acid (6.6 mmol, 924 mg) with a phosphorus pentoxide—methanesulfonic acid mixture (P_2_O_5_—MsOH [1/10 *w*/*w*] 13.2 ml). After the reaction mixture had been stirred at 333 K for 1 h, the mixture was poured into ice-cold water and extracted with CHCl_3_. The organic layer thus obtained was dried over anhydrous MgSO_4_. The solvent was removed under reduced pressure to give a cake. The crude product was purified by recrystallization from CHCl_3_– hexane (*v*/*v* = 1:2) [39% isolated yield; M.p. 364.5–366 K]. HRMS (*m/z*): [*M* + H]^+^ calcd. for C_32_H_31_F_2_O_4_, 517.2190; found 517.2163. Block-like colourless crystals of the title compound, suitable for X-ray diffraction analysis, were obtained by crystallization from hexane. Spectroscopic data for the title compound are available in the archived CIF.

### Refinement

All the H atoms were included in calculated positions and treated as riding on their parent atoms: C—H = 0.95 (aromatic ), 0.98 (methyl), 0.99 (methylene) Å, with *U*_iso_(H) = 1.2*U*_eq_(C). The positions of methyl H atoms were rotationally optimized.

### Crystal data

**Table:**

C_32_H_30_F_2_O_4_	*F*(000) = 1088
*M**_r_* = 516.56	*D*_x_ = 1.261 Mg m^−^^3^
Monoclinic, *P*2_1_/*c*	Cu *K*α radiation, λ = 1.54187 Å
Hall symbol: -P 2ybc	Cell parameters from 45409 reflections
*a* = 8.26012 (15) Å	θ = 3.5–68.2°
*b* = 20.2309 (4) Å	µ = 0.75 mm^−^^1^
*c* = 16.5268 (3) Å	*T* = 193 K
β = 99.918 (1)°	Block, colourless
*V* = 2720.51 (9) Å^3^	0.60 × 0.50 × 0.50 mm
*Z* = 4	

### Data collection

**Table:**

Rigaku R-AXIS RAPID diffractometer	4971 independent reflections
Radiation source: rotating anode	4715 reflections with *I* > 2σ(*I*)
Graphite monochromator	*R*_int_ = 0.047
Detector resolution: 10.000 pixels mm^-1^	θ_max_ = 68.2°, θ_min_ = 3.5°
ω scans	*h* = −9→9
Absorption correction: numerical (*NUMABS*; Higashi, 1999)	*k* = −24→24
*T*_min_ = 0.661, *T*_max_ = 0.705	*l* = −19→19
50395 measured reflections	

### Refinement

**Table:**

Refinement on *F*^2^	Secondary atom site location: difference Fourier map
Least-squares matrix: full	Hydrogen site location: inferred from neighbouring sites
*R*[*F*^2^ > 2σ(*F*^2^)] = 0.045	H-atom parameters constrained
*wR*(*F*^2^) = 0.116	*w* = 1/[σ^2^(*F*_o_^2^) + (0.0573*P*)^2^ + 1.0814*P*] where *P* = (*F*_o_^2^ + 2*F*_c_^2^)/3
*S* = 1.03	(Δ/σ)_max_ < 0.001
4971 reflections	Δρ_max_ = 0.35 e Å^−^^3^
346 parameters	Δρ_min_ = −0.41 e Å^−^^3^
0 restraints	Extinction correction: *SHELXL97* (Sheldrick, 2008), Fc^*^=kFc[1+0.001xFc^2^λ^3^/sin(2θ)]^-1/4^
Primary atom site location: structure-invariant direct methods	Extinction coefficient: 0.0198 (6)

### Special details

**Table:**

Experimental. Spectroscopic data for the title compound:^1^HNMR δ (300 MHz, CDCl_3_): 0.68 (6*H*, t, *J*=7.5 Hz), 0.90–1.02 (4*H*, m), 1.27–1.36 (4*H*, m) 3.89 (4*H*, t, J=6.1 Hz), 7.04 (4*H*, t, J=8.5 Hz), 7.15 (2*H*, d, J=8.9 Hz), 7.75 (4*H*, dd, J=8.5, 5.1 Hz), 7.91 (2*H*, d, J=8.9 Hz) p.p.m.^13^CNMR δ (75 MHz, CDCl_3_): 13.49, 18.63, 30.93, 68.55, 111.63, 114.93 (d, ^2^*J*_C—F_=21.6 Hz), 120.78, 125.28, 130.33, 131.49 (d, ^3^*J*_C—F_=7.9 Hz), 132.23, 135.68(d, ^4^*J*_C—F_=2.8 Hz), 155.94, 165.45(d, ^1^*J*_C—F_=253.5 Hz), 196.16 p.p.m.IR (KBr): 1659 (C═O), 1595, 1508, 1466 (Ar, naphthalene), 1236 (═C—O—C) cm^-1^.
Geometry. All e.s.d.'s (except the e.s.d. in the dihedral angle between two l.s. planes) are estimated using the full covariance matrix. The cell e.s.d.'s are taken into account individually in the estimation of e.s.d.'s in distances, angles and torsion angles; correlations between e.s.d.'s in cell parameters are only used when they are defined by crystal symmetry. An approximate (isotropic) treatment of cell e.s.d.'s is used for estimating e.s.d.'s involving l.s. planes.
Refinement. Refinement of *F*^2^ against ALL reflections. The weighted *R*-factor *wR* and goodness of fit *S* are based on *F*^2^, conventional *R*-factors *R* are based on *F*, with *F* set to zero for negative *F*^2^. The threshold expression of *F*^2^ > σ(*F*^2^) is used only for calculating *R*-factors(gt) *etc*. and is not relevant to the choice of reflections for refinement. *R*-factors based on *F*^2^ are statistically about twice as large as those based on *F*, and *R*- factors based on ALL data will be even larger.

### Fractional atomic coordinates and isotropic or equivalent isotropic displacement parameters (Å^2^)

**Table:**

	*x*	*y*	*z*	*U*_iso_*/*U*_eq_	
F1	1.2676 (2)	0.02657 (7)	0.89453 (9)	0.1074 (6)	
F2	0.04618 (12)	−0.02158 (5)	0.58323 (6)	0.0579 (3)	
O1	0.56247 (13)	0.14733 (5)	0.77614 (7)	0.0455 (3)	
O2	0.72484 (11)	0.12311 (5)	0.61173 (6)	0.0383 (3)	
O3	0.84151 (15)	0.25811 (5)	0.87922 (6)	0.0486 (3)	
O4	0.44905 (13)	0.20244 (5)	0.46369 (6)	0.0405 (3)	
C1	0.71284 (15)	0.24026 (6)	0.74515 (8)	0.0313 (3)	
C2	0.78418 (17)	0.28485 (7)	0.80412 (9)	0.0353 (3)	
C3	0.79004 (17)	0.35302 (7)	0.78743 (9)	0.0381 (3)	
H3	0.8429	0.3827	0.8281	0.046*	
C4	0.71875 (17)	0.37570 (7)	0.71201 (9)	0.0376 (3)	
H4	0.7208	0.4218	0.7010	0.045*	
C5	0.56894 (17)	0.35703 (7)	0.57188 (10)	0.0392 (3)	
H5	0.5684	0.4033	0.5624	0.047*	
C6	0.49959 (18)	0.31636 (7)	0.51018 (9)	0.0393 (3)	
H6	0.4501	0.3340	0.4586	0.047*	
C7	0.50210 (16)	0.24734 (7)	0.52385 (8)	0.0335 (3)	
C8	0.56831 (15)	0.22084 (6)	0.59952 (8)	0.0301 (3)	
C9	0.64011 (15)	0.26306 (6)	0.66526 (8)	0.0298 (3)	
C10	0.64187 (16)	0.33263 (7)	0.64976 (9)	0.0338 (3)	
C11	0.69783 (18)	0.17029 (7)	0.77425 (8)	0.0347 (3)	
C12	0.8510 (2)	0.13180 (7)	0.80393 (9)	0.0406 (4)	
C13	0.8421 (3)	0.07733 (8)	0.85437 (11)	0.0577 (5)	
H13	0.7398	0.0643	0.8682	0.069*	
C14	0.9857 (4)	0.04188 (9)	0.88456 (13)	0.0755 (7)	
H14	0.9821	0.0048	0.9194	0.091*	
C15	1.1306 (3)	0.06148 (10)	0.86313 (13)	0.0699 (6)	
C16	1.1431 (2)	0.11352 (10)	0.81259 (12)	0.0608 (5)	
H16	1.2455	0.1251	0.7977	0.073*	
C17	1.0016 (2)	0.14922 (8)	0.78338 (10)	0.0462 (4)	
H17	1.0078	0.1862	0.7487	0.055*	
C18	0.58717 (16)	0.14675 (6)	0.60404 (8)	0.0302 (3)	
C19	0.43961 (16)	0.10364 (6)	0.59768 (8)	0.0314 (3)	
C20	0.45880 (19)	0.03641 (7)	0.58633 (10)	0.0414 (3)	
H20	0.5643	0.0193	0.5825	0.050*	
C21	0.3262 (2)	−0.00605 (8)	0.58051 (11)	0.0492 (4)	
H21	0.3386	−0.0520	0.5717	0.059*	
C22	0.17610 (19)	0.02009 (8)	0.58782 (9)	0.0419 (4)	
C23	0.15121 (18)	0.08624 (8)	0.59960 (9)	0.0413 (3)	
H23	0.0456	0.1027	0.6045	0.050*	
C24	0.28524 (17)	0.12828 (7)	0.60418 (9)	0.0369 (3)	
H24	0.2714	0.1743	0.6118	0.044*	
C25	0.88713 (18)	0.29933 (7)	0.94971 (9)	0.0382 (3)	
H25A	0.7952	0.3289	0.9569	0.046*	
H25B	0.9836	0.3268	0.9438	0.046*	
C26	0.92802 (18)	0.25342 (8)	1.02206 (9)	0.0398 (3)	
H26A	0.8301	0.2261	1.0257	0.048*	
H26B	0.9522	0.2803	1.0728	0.048*	
C27	1.07163 (19)	0.20804 (9)	1.01883 (10)	0.0479 (4)	
H27A	1.1707	0.2349	1.0162	0.057*	
H27B	1.0486	0.1810	0.9682	0.057*	
C28	1.1057 (3)	0.16282 (13)	1.09237 (14)	0.0802 (7)	
H28A	1.0070	0.1369	1.0961	0.096*	
H28B	1.1359	0.1893	1.1424	0.096*	
H28C	1.1963	0.1329	1.0864	0.096*	
C29	0.3849 (2)	0.22518 (8)	0.38231 (9)	0.0450 (4)	
H29A	0.4614	0.2573	0.3640	0.054*	
H29B	0.2775	0.2471	0.3812	0.054*	
C30	0.3653 (2)	0.16535 (9)	0.32649 (10)	0.0513 (4)	
H30A	0.3280	0.1805	0.2693	0.062*	
H30B	0.4743	0.1444	0.3288	0.062*	
C31	0.2476 (2)	0.11429 (10)	0.34659 (12)	0.0576 (5)	
H31A	0.1405	0.1356	0.3485	0.069*	
H31B	0.2895	0.0961	0.4018	0.069*	
C32	0.2216 (3)	0.05793 (10)	0.28513 (12)	0.0640 (5)	
H32A	0.3284	0.0404	0.2773	0.077*	
H32B	0.1616	0.0742	0.2325	0.077*	
H32C	0.1579	0.0228	0.3059	0.077*	

### Atomic displacement parameters (Å^2^)

**Table:**

	*U*^11^	*U*^22^	*U*^33^	*U*^12^	*U*^13^	*U*^23^
F1	0.1195 (12)	0.0818 (9)	0.0989 (10)	0.0636 (9)	−0.0436 (9)	−0.0190 (8)
F2	0.0509 (6)	0.0487 (5)	0.0696 (7)	−0.0224 (4)	−0.0023 (5)	0.0128 (5)
O1	0.0498 (6)	0.0446 (6)	0.0423 (6)	−0.0115 (5)	0.0087 (5)	−0.0009 (5)
O2	0.0314 (5)	0.0375 (5)	0.0448 (6)	0.0028 (4)	0.0034 (4)	−0.0010 (4)
O3	0.0672 (7)	0.0353 (5)	0.0363 (6)	0.0048 (5)	−0.0103 (5)	−0.0082 (4)
O4	0.0463 (6)	0.0388 (5)	0.0322 (5)	−0.0041 (4)	−0.0056 (4)	0.0028 (4)
C1	0.0283 (6)	0.0300 (7)	0.0351 (7)	0.0008 (5)	0.0040 (5)	−0.0022 (5)
C2	0.0325 (7)	0.0353 (7)	0.0367 (7)	0.0024 (5)	0.0017 (6)	−0.0040 (6)
C3	0.0348 (7)	0.0335 (7)	0.0450 (8)	−0.0030 (6)	0.0040 (6)	−0.0093 (6)
C4	0.0354 (7)	0.0281 (7)	0.0494 (8)	−0.0024 (5)	0.0082 (6)	−0.0016 (6)
C5	0.0373 (7)	0.0292 (7)	0.0501 (9)	−0.0004 (6)	0.0048 (6)	0.0070 (6)
C6	0.0354 (7)	0.0390 (8)	0.0410 (8)	−0.0004 (6)	−0.0008 (6)	0.0101 (6)
C7	0.0267 (6)	0.0365 (7)	0.0360 (7)	−0.0029 (5)	0.0016 (5)	0.0016 (6)
C8	0.0250 (6)	0.0307 (7)	0.0343 (7)	−0.0020 (5)	0.0043 (5)	0.0012 (5)
C9	0.0238 (6)	0.0306 (7)	0.0350 (7)	−0.0009 (5)	0.0054 (5)	−0.0002 (5)
C10	0.0288 (6)	0.0309 (7)	0.0421 (8)	−0.0007 (5)	0.0073 (6)	0.0008 (6)
C11	0.0439 (8)	0.0330 (7)	0.0262 (6)	−0.0036 (6)	0.0030 (5)	−0.0040 (5)
C12	0.0572 (9)	0.0293 (7)	0.0307 (7)	0.0032 (6)	−0.0050 (6)	−0.0053 (6)
C13	0.0858 (13)	0.0362 (8)	0.0443 (9)	−0.0033 (8)	−0.0083 (9)	0.0017 (7)
C14	0.124 (2)	0.0338 (9)	0.0534 (11)	0.0130 (11)	−0.0264 (12)	0.0031 (8)
C15	0.0836 (15)	0.0515 (11)	0.0615 (12)	0.0295 (11)	−0.0238 (11)	−0.0164 (9)
C16	0.0592 (11)	0.0634 (12)	0.0534 (10)	0.0226 (9)	−0.0078 (8)	−0.0186 (9)
C17	0.0502 (9)	0.0459 (9)	0.0392 (8)	0.0112 (7)	−0.0014 (7)	−0.0071 (7)
C18	0.0312 (7)	0.0328 (7)	0.0253 (6)	0.0001 (5)	0.0012 (5)	−0.0007 (5)
C19	0.0347 (7)	0.0310 (7)	0.0268 (6)	−0.0023 (5)	0.0002 (5)	0.0008 (5)
C20	0.0403 (8)	0.0338 (7)	0.0491 (9)	0.0004 (6)	0.0046 (6)	−0.0003 (6)
C21	0.0551 (10)	0.0290 (7)	0.0616 (10)	−0.0069 (7)	0.0044 (8)	−0.0001 (7)
C22	0.0418 (8)	0.0389 (8)	0.0416 (8)	−0.0143 (6)	−0.0025 (6)	0.0077 (6)
C23	0.0332 (7)	0.0435 (8)	0.0455 (8)	−0.0037 (6)	0.0025 (6)	0.0066 (6)
C24	0.0355 (7)	0.0315 (7)	0.0422 (8)	−0.0007 (6)	0.0029 (6)	0.0014 (6)
C25	0.0406 (8)	0.0385 (8)	0.0357 (7)	−0.0051 (6)	0.0067 (6)	−0.0106 (6)
C26	0.0393 (8)	0.0463 (8)	0.0343 (7)	−0.0035 (6)	0.0076 (6)	−0.0082 (6)
C27	0.0377 (8)	0.0635 (10)	0.0422 (8)	0.0031 (7)	0.0059 (6)	0.0009 (7)
C28	0.0728 (14)	0.1043 (18)	0.0640 (13)	0.0279 (13)	0.0127 (10)	0.0292 (12)
C29	0.0511 (9)	0.0500 (9)	0.0313 (7)	−0.0034 (7)	0.0001 (6)	0.0089 (6)
C30	0.0527 (9)	0.0664 (11)	0.0341 (8)	−0.0017 (8)	0.0054 (7)	0.0011 (7)
C31	0.0510 (10)	0.0684 (12)	0.0541 (10)	−0.0076 (8)	0.0113 (8)	−0.0129 (9)
C32	0.0810 (13)	0.0589 (11)	0.0475 (10)	−0.0034 (10)	−0.0018 (9)	−0.0066 (8)

### Geometric parameters (Å, º)

**Table:**

F1—C15	1.359 (2)	C18—C19	1.4876 (18)
F2—C22	1.3566 (16)	C19—C20	1.386 (2)
O1—C11	1.2160 (18)	C19—C24	1.3904 (19)
O2—C18	1.2199 (16)	C20—C21	1.382 (2)
O3—C2	1.3620 (17)	C20—H20	0.9500
O3—C25	1.4294 (16)	C21—C22	1.372 (2)
O4—C7	1.3622 (17)	C21—H21	0.9500
O4—C29	1.4337 (17)	C22—C23	1.373 (2)
C1—C2	1.3830 (19)	C23—C24	1.388 (2)
C1—C9	1.4297 (19)	C23—H23	0.9500
C1—C11	1.5070 (19)	C24—H24	0.9500
C2—C3	1.409 (2)	C25—C26	1.506 (2)
C3—C4	1.363 (2)	C25—H25A	0.9900
C3—H3	0.9500	C25—H25B	0.9900
C4—C10	1.413 (2)	C26—C27	1.508 (2)
C4—H4	0.9500	C26—H26A	0.9900
C5—C6	1.358 (2)	C26—H26B	0.9900
C5—C10	1.413 (2)	C27—C28	1.508 (3)
C5—H5	0.9500	C27—H27A	0.9900
C6—C7	1.414 (2)	C27—H27B	0.9900
C6—H6	0.9500	C28—H28A	0.9800
C7—C8	1.3839 (19)	C28—H28B	0.9800
C8—C9	1.4281 (18)	C28—H28C	0.9800
C8—C18	1.5076 (18)	C29—C30	1.514 (2)
C9—C10	1.4312 (19)	C29—H29A	0.9900
C11—C12	1.495 (2)	C29—H29B	0.9900
C12—C17	1.390 (2)	C30—C31	1.495 (3)
C12—C13	1.391 (2)	C30—H30A	0.9900
C13—C14	1.402 (3)	C30—H30B	0.9900
C13—H13	0.9500	C31—C32	1.517 (2)
C14—C15	1.365 (4)	C31—H31A	0.9900
C14—H14	0.9500	C31—H31B	0.9900
C15—C16	1.359 (3)	C32—H32A	0.9800
C16—C17	1.388 (2)	C32—H32B	0.9800
C16—H16	0.9500	C32—H32C	0.9800
C17—H17	0.9500		
			
C2—O3—C25	120.83 (11)	C19—C20—H20	119.6
C7—O4—C29	119.42 (11)	C22—C21—C20	118.14 (14)
C2—C1—C9	120.04 (12)	C22—C21—H21	120.9
C2—C1—C11	115.87 (12)	C20—C21—H21	120.9
C9—C1—C11	123.58 (12)	F2—C22—C21	118.27 (14)
O3—C2—C1	114.97 (12)	F2—C22—C23	118.52 (14)
O3—C2—C3	123.29 (13)	C21—C22—C23	123.22 (14)
C1—C2—C3	121.69 (13)	C22—C23—C24	117.88 (14)
C4—C3—C2	118.98 (13)	C22—C23—H23	121.1
C4—C3—H3	120.5	C24—C23—H23	121.1
C2—C3—H3	120.5	C23—C24—C19	120.64 (13)
C3—C4—C10	121.83 (13)	C23—C24—H24	119.7
C3—C4—H4	119.1	C19—C24—H24	119.7
C10—C4—H4	119.1	O3—C25—C26	106.19 (12)
C6—C5—C10	122.08 (13)	O3—C25—H25A	110.5
C6—C5—H5	119.0	C26—C25—H25A	110.5
C10—C5—H5	119.0	O3—C25—H25B	110.5
C5—C6—C7	119.06 (13)	C26—C25—H25B	110.5
C5—C6—H6	120.5	H25A—C25—H25B	108.7
C7—C6—H6	120.5	C25—C26—C27	114.69 (12)
O4—C7—C8	115.38 (12)	C25—C26—H26A	108.6
O4—C7—C6	123.22 (12)	C27—C26—H26A	108.6
C8—C7—C6	121.31 (13)	C25—C26—H26B	108.6
C7—C8—C9	120.23 (12)	C27—C26—H26B	108.6
C7—C8—C18	116.83 (12)	H26A—C26—H26B	107.6
C9—C8—C18	122.08 (11)	C26—C27—C28	112.29 (15)
C8—C9—C1	124.26 (12)	C26—C27—H27A	109.1
C8—C9—C10	117.89 (12)	C28—C27—H27A	109.1
C1—C9—C10	117.84 (12)	C26—C27—H27B	109.1
C4—C10—C5	121.04 (13)	C28—C27—H27B	109.1
C4—C10—C9	119.56 (13)	H27A—C27—H27B	107.9
C5—C10—C9	119.39 (13)	C27—C28—H28A	109.5
O1—C11—C12	121.51 (13)	C27—C28—H28B	109.5
O1—C11—C1	119.57 (13)	H28A—C28—H28B	109.5
C12—C11—C1	118.86 (12)	C27—C28—H28C	109.5
C17—C12—C13	119.27 (16)	H28A—C28—H28C	109.5
C17—C12—C11	122.02 (14)	H28B—C28—H28C	109.5
C13—C12—C11	118.71 (16)	O4—C29—C30	107.38 (13)
C12—C13—C14	119.3 (2)	O4—C29—H29A	110.2
C12—C13—H13	120.3	C30—C29—H29A	110.2
C14—C13—H13	120.3	O4—C29—H29B	110.2
C15—C14—C13	119.01 (19)	C30—C29—H29B	110.2
C15—C14—H14	120.5	H29A—C29—H29B	108.5
C13—C14—H14	120.5	C31—C30—C29	115.01 (14)
C16—C15—F1	119.4 (2)	C31—C30—H30A	108.5
C16—C15—C14	123.20 (18)	C29—C30—H30A	108.5
F1—C15—C14	117.4 (2)	C31—C30—H30B	108.5
C15—C16—C17	117.9 (2)	C29—C30—H30B	108.5
C15—C16—H16	121.0	H30A—C30—H30B	107.5
C17—C16—H16	121.0	C30—C31—C32	113.15 (16)
C16—C17—C12	121.23 (17)	C30—C31—H31A	108.9
C16—C17—H17	119.4	C32—C31—H31A	108.9
C12—C17—H17	119.4	C30—C31—H31B	108.9
O2—C18—C19	121.02 (12)	C32—C31—H31B	108.9
O2—C18—C8	118.77 (12)	H31A—C31—H31B	107.8
C19—C18—C8	120.20 (11)	C31—C32—H32A	109.5
C20—C19—C24	119.35 (13)	C31—C32—H32B	109.5
C20—C19—C18	118.26 (13)	H32A—C32—H32B	109.5
C24—C19—C18	122.38 (12)	C31—C32—H32C	109.5
C21—C20—C19	120.76 (14)	H32A—C32—H32C	109.5
C21—C20—H20	119.6	H32B—C32—H32C	109.5
			
C25—O3—C2—C1	−167.14 (12)	O1—C11—C12—C17	163.03 (14)
C25—O3—C2—C3	10.4 (2)	C1—C11—C12—C17	−19.90 (19)
C9—C1—C2—O3	176.55 (12)	O1—C11—C12—C13	−17.7 (2)
C11—C1—C2—O3	4.41 (18)	C1—C11—C12—C13	159.41 (13)
C9—C1—C2—C3	−1.0 (2)	C17—C12—C13—C14	1.1 (2)
C11—C1—C2—C3	−173.19 (13)	C11—C12—C13—C14	−178.22 (15)
O3—C2—C3—C4	−175.24 (13)	C12—C13—C14—C15	−0.5 (3)
C1—C2—C3—C4	2.2 (2)	C13—C14—C15—C16	−1.0 (3)
C2—C3—C4—C10	−1.2 (2)	C13—C14—C15—F1	179.03 (15)
C10—C5—C6—C7	−0.8 (2)	F1—C15—C16—C17	−178.29 (15)
C29—O4—C7—C8	−177.39 (12)	C14—C15—C16—C17	1.7 (3)
C29—O4—C7—C6	−0.9 (2)	C15—C16—C17—C12	−1.0 (2)
C5—C6—C7—O4	−173.93 (13)	C13—C12—C17—C16	−0.4 (2)
C5—C6—C7—C8	2.4 (2)	C11—C12—C17—C16	178.95 (14)
O4—C7—C8—C9	174.85 (11)	C7—C8—C18—O2	108.25 (14)
C6—C7—C8—C9	−1.7 (2)	C9—C8—C18—O2	−61.18 (17)
O4—C7—C8—C18	5.22 (17)	C7—C8—C18—C19	−70.66 (16)
C6—C7—C8—C18	−171.35 (12)	C9—C8—C18—C19	119.90 (13)
C7—C8—C9—C1	−179.36 (12)	O2—C18—C19—C20	−11.03 (19)
C18—C8—C9—C1	−10.28 (19)	C8—C18—C19—C20	167.86 (13)
C7—C8—C9—C10	−0.46 (18)	O2—C18—C19—C24	167.79 (13)
C18—C8—C9—C10	168.62 (12)	C8—C18—C19—C24	−13.32 (19)
C2—C1—C9—C8	177.88 (12)	C24—C19—C20—C21	0.7 (2)
C11—C1—C9—C8	−10.6 (2)	C18—C19—C20—C21	179.59 (14)
C2—C1—C9—C10	−1.01 (18)	C19—C20—C21—C22	−1.2 (2)
C11—C1—C9—C10	170.50 (12)	C20—C21—C22—F2	−179.14 (14)
C3—C4—C10—C5	−179.85 (13)	C20—C21—C22—C23	0.8 (3)
C3—C4—C10—C9	−0.9 (2)	F2—C22—C23—C24	179.99 (13)
C6—C5—C10—C4	177.59 (14)	C21—C22—C23—C24	0.1 (2)
C6—C5—C10—C9	−1.4 (2)	C22—C23—C24—C19	−0.5 (2)
C8—C9—C10—C4	−177.00 (12)	C20—C19—C24—C23	0.2 (2)
C1—C9—C10—C4	1.96 (18)	C18—C19—C24—C23	−178.65 (13)
C8—C9—C10—C5	1.97 (18)	C2—O3—C25—C26	174.59 (12)
C1—C9—C10—C5	−179.06 (12)	O3—C25—C26—C27	62.58 (16)
C2—C1—C11—O1	112.36 (15)	C25—C26—C27—C28	−179.43 (16)
C9—C1—C11—O1	−59.48 (18)	C7—O4—C29—C30	170.00 (12)
C2—C1—C11—C12	−64.77 (16)	O4—C29—C30—C31	61.91 (19)
C9—C1—C11—C12	123.39 (14)	C29—C30—C31—C32	175.40 (16)

### Hydrogen-bond geometry (Å, º)

Cg1 and Cg2 are the centroids of the C12–C17 and C5–C10 rings, respectively.

**Table:**

*D*—H···*A*	*D*—H	H···*A*	*D*···*A*	*D*—H···*A*
C27—H27*B*···*Cg*1	0.99	2.79	3.7754 (19)	175
C26—H26*A*···*Cg*2^i^	0.99	2.54	3.4239 (16)	145
C3—H3···F2^ii^	0.95	2.50	3.4408 (17)	169

Symmetry codes: (i) *x*, −*y*+1/2, *z*+1/2; (ii) −*x*+1, *y*+1/2, −*z*+3/2.
